# Endosymbiotic Calcifying Bacteria: A New Cue to the Origin of Calcification in Metazoa?

**DOI:** 10.1111/j.1558-5646.2012.01676.x

**Published:** 2012-10

**Authors:** Maria J Uriz, Gemma Agell, Andrea Blanquer, Xavier Turon, Emilio O Casamayor

**Affiliations:** 1 Centre d’Estudis Avançats de Blanes, CEAB-CSIC. Accés Cala St Francesc14#17300 Blanes (Girona), Spain; 3 UPMC Univ Paris 06, Observatoire OcéanologiqueF-66650, Banyuls/Mer, France; CNRS, FRE 3350; 4 Laboratoire d'écogéochimie des environnements benthiques (LECOB), Observatoire OcéanologiqueF-66650, Banyuls/Mer, France

**Keywords:** Biomineralization, calcibacteria, marine sponges, symbiosis

## Abstract

Sponges show the highest diversity of associated bacteria among marine invertebrates. Immunological evidence traces the origin of the sponge bacterial symbioses to the Precambrian era. Hence, sponges appear to be ideally suited for studying the evolutionary origins of prokaryote–metazoan associations. Sponges produce either calcareous or siliceous skeletons, which only coexist in a relict group of demosponges, the sclerosponges. We report here, for the first time, intensive calcification in nonsclerosponge siliceous demosponges. Calcification is mediated by endosymbiotic bacteria (calcibacteria) located in archeocyte-like sponge cells. These calcibacteria are devoid of bacterial walls and divide within sponge cells until they became surrounded by a calcitic sheet, being subsequently extruded to the sponge subectosomal (subepithelial) zone. Thousands of bacteria-produced calcitic spherules cover the surface of the host sponges, forming a cortex-like structure that mimics a rudimentary peripheral skeleton. Calcibacteria are vertically transferred to the sponge larvae during embryogenesis. Calcium detoxification may have generated this symbiotic association, with some additional benefits for the sponges, such as skeletal formation and deterrence from predation. This unique symbiosis holds implications for sponge biology and may advance discussions on the role of bacteria in early biocalcification processes in metazoans.

Sponges (Porifera) are the earliest branching metazoans (Li et al. 1998; Philippe et al. 2009), and they show the highest diversity of associated bacteria among marine invertebrates (Schmitt et al. 2011). Immunological evidence has tracked the origin of sponge bacterial symbioses to before the Cambrian period (Wilkinson 1984). Therefore, sponges appear to be appropriate organisms for studying the evolutionary origins of prokaryote–metazoan symbioses.

Extant sponges have either siliceous or calcareous skeletons (Uriz 2006), which rarely coexist in the same species. So far, the only exception to this clear-cut dichotomy in the mineral nature of sponge skeletons is the so-called coralline demosponges or sclerosponges that produce a massive calcium carbonate skeleton and free siliceous spicules simultaneously (Vacelet 1985). Extant sclerosponges are considered relict, but representatives of this group proliferated and built reefs in the late Permian (Paleozoic) and late Triassic (Mesozoic) periods (Reitner 1989). The adaptive potential of a double mineralization is illustrated by examples of extant sclerosponges (e.g., genus *Merlia*). This genus usually shows both types of mineral skeletons, but produces mainly a calcareous skeleton in silica-depleted habitats (i.e., *M. lipoclavidisca*Vacelet and Uriz 1991) or mainly a siliceous skeleton in silica-rich habitats (i.e., *M. deficiens*Vacelet 1980). The calcareous skeletons of sclerosponges are made of intracellular spherical structures (spherulites) that are excreted to the sponge mesohyl, where they grow epitaxially, by successive superposition of aragonite-made layers, to build walls and chambers (Willenz and Hartman 1989; Wood 1991; Wörheide 1998). The possible role of symbiotic bacteria in the formation of the sclerosponge spherulites has been considered. A bacterial signature was found in the spherulites of the sclerosponge *Astrosclera willeyana* (Jackson et al. 2010). Later, Jackson et al. (2011) reported the presence of a protein (spheruline) in the spherulites of this species, which, according to several lines of evidence (Jackson et al. 2011), seems likely to be encoded by a sponge gene horizontally acquired from a bacterium. Thus, there is growing indication of the participation of prokaryote genes in sponge biocalcification, but the direct involvement of symbiotic bacteria has never been proved.

Here, we present the first evidence of endosymbiotic calcifying bacteria (hereafter calcibacteria) within nonsclerosponge sponges of the genus *Hemimycale* (Demospongiae: Poecilosclerida, Van Soest 2002). These calcifying bacteria may have implications for sponge biology and may support the hypothesis of an early endosymbiotic origin of calcification in metazoans.

## Methods

### SAMPLING AND EXPERIMENTAL PROCEDURES

Fresh sponges of the genus *Hemimycale* were collected from several seas (*H. columella*: NE Spain, Mediterranean Sea; *H. arabica*: Red Sea, *Hemimycale* sp.: E Africa, Indian Ocean) and immediately processed for light, epifluorescence Transmission Electron (TEM) and Scanning Electron (SEM) microscopy studies, and Transmission Electron Microscope (TEM) and Scanning Electron Microscope (SEM). The presence of calcibacteria was microscopically verified less than 1 h after collection.

### CALCIBACTERIA QUANTIFICATION

To determine the amount of calcium carbonate (as a proxy for calcibacteria abundance), specimens (*N*= 5 per species) were dried at 80 °C for 48 h in a stove and then weighed (A) using an analytical balance. The dried samples were placed in an oven at 600 °C to remove the organic matter. The remaining minerals, mainly consisting of siliceous spicules and calcibacterial coats, were weighed (B) and put in a Pyrex fingerbowl, where the calcareous material was removed through boiling in nitric acid. This process left a residue mostly consisting of siliceous spicules that were rinsed with distilled water and dehydrated with absolute ethanol before being dried and weighed (C). The weight of the calcibacteria coats was calculated as B–C and was referred to as the sponge dry weight in percentage by the formula (B–C) × 100/A.

### MORPHOLOGICAL AND ULTRASTRUCTURE OBSERVATION (TEM AND SEM)

Small pieces, approximately 3 mm in diameter, were obtained from living sponges (*H. columella, H. arabica*), fixed for TEM (Uriz et al. 2008), and embedded in Spurr. Ultrathin sections were lead citrate stained and observed through a JEOL JEM-1010 TEM (Scientific-Technical Services of the University of Barcelona). Isolated calcibacteria were dehydrated, gold palladium metalized, and observed through a Hitachi S-3520N SEM (Microscopy Service ICM-CSIC, Barcelona). Calcibacteria cryofracture was performed in liquid nitrogen to observe the inner part of the calcibacterial coat. For light microscope observation, fresh samples were dissociated in seawater by stirring them with a pipette and a drop of cell suspension was observed.

### ENERGY-DISPERSIVE X-RAY (EDX) ANALYSIS

EDX analysis was performed to determine the chemical nature of the calcibacterial coat (RÖNTEC Microanalyzer ICM-CSIC, Barcelona) according to standard practice (Turon et al. 2000).

### DAPI AND FISH

DNA staining and in situ hybridization with bacterial and archaeal probes was performed on sponge sections. A fragment of a living individual of *H. columella* was fixed in 4% buffered paraformaldehyde (PANREAC, Spain). This fragment was dehydrated and embedded in paraffin, cut into 5-μm-thick sections, mounted on slides and stored at −20 °C. Sponge sections were DAPI stained and used for catalyzed-reported deposition whole cell fluorescence in situ hybridization (CARD-FISH) with bacteria-specific (EUB338) and archaea-specific (ARC915) probes (Pernthaler and Amann 2004; Auguet and Casamayor 2008). Other bacterial probes were also assayed for Betaproteobacteria (BET42a), Bacteroidetes (CF319a), and Actinobacteria (HGC69a), but they gave negative results. All probes were synthetized by Molecular Probes (Invitrogen, USA). The hybridized DNA was observed with a Zeiss epifluorescence microscope using the appropriate filter sets for DAPI (Zeiss filter set 01, BP365/12 FT396 LP397) and Alexa-Fluor 488 (Zeiss filter set 09, BP450–490 FT510 LP515).

## Results

**Figure 1:**
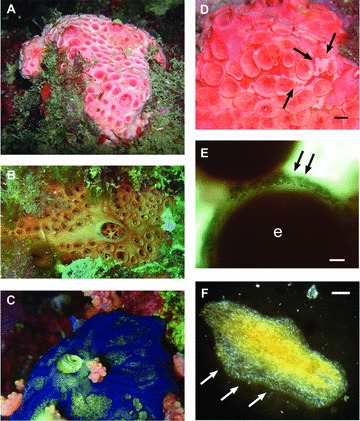
Sponge species harboring calcibacteria. (A) *Hemimycale columella* (Atlanto-Mediterranean). (B) *Hemimycale* sp. (Indo-Pacific). (C) *Hemimycale arabica* (Red Sea). (D) Whitish tinge of the sponge surface (arrows) due to calcibacteria accumulation; scale bar 2 mm. (E) Calcibacteriocytes (arrows) surrounding an embryo; (e) scale bar 50 μm. (F) Calcibacteria accumulation (white spots) in a 2-week-old recruit (rhagon); scale bar 1 mm.

Sponge calcibacteria were extraordinarily abundant (their calcareous coats represented 30–60% of the sponge dry weight) in all three currently known species of the genus *Hemimycale* (Demospongiae: Poecilosclerida) inhabiting different seas, that is, *H. columella* (Bowerbank 1874), Atlanto-Mediterranean; *H. arabica* (Ilan et al. 2004), Red Sea; and *Hemimycale* sp. (taxonomic status currently being determined by the authors), Indian Ocean, E. Africa (Fig. 1 A–C).

**Figure 2:**
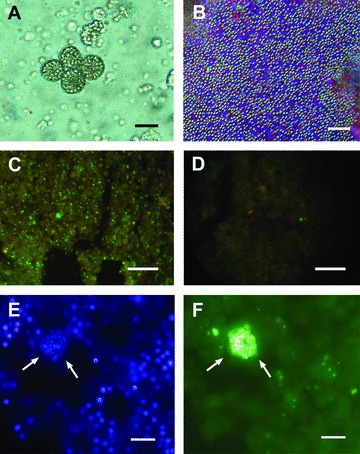
Light microscopy pictures of calcibacteriocytes and calcibacteria. (A) Calcibacteriocytes full of refringent calcibacteria; scale bar 5 μm. (B) Released calcibacteria after crushing a living individual of *Hemimycale columella*; scale bar 10 μm. (C, D) CARD-FISH epifluorescence micrographs of *H. columella* sections, either labeled with bacteria-specific probe EUB338 (green) (C) or with archaea-specific probe ARCH915 (D); scale bar 40 μm. (E, F) Higher magnification of sponge section showing a calcibacteria-full calcibacteriocyte (arrows) either stained with DAPI (E)—see also sponge cell nuclei (n)—or labeled with bacteria-specific probe; (F) scale bar 5 μm.

The calcibacteria were coccoid in shape and 200–800 nm in diameter (about 500 nm on average) and were found within vacuoles of amoeboid, archeocyte-like sponge cells (hereafter calcibacteriocytes) of about 15 μm in diameter (Fig. 2A, B). Degenerated calcibacteria were also observed in the sponge mesohyl as a result of calcibacteriocyte lysis. The bacterial nature of the calcibacteria was confirmed by electron (TEM) and epifluorescence microscope observations (DAPI staining and CARD-FISH techniques with specific prokaryote probes). Calcibacteria showed positive hybridization with the universal eubacterial probe (Fig. 2C, F) and only rare signals with the universal archaeal probe (Fig. 2D).

**Figure 3:**
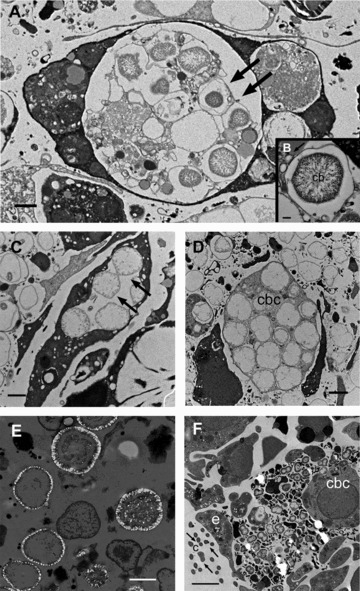
TEM micrographs. (A) Early calcibacteria (arrows) within calcibacteriocyte vacuoles; scale bar 1μm. (B) Calcibacteria (cb) starting biocalcification (arrow points to calcification vesicles); scale bar 0.2 μm. (C) Calcibacteria division within a vacuole (arrows); scale bar 1 μm. (D) Calcibacteriocyte (cbc) with dividing calcified bacteria; scale bar 1 μm. The bacteria calcareous coat is not stained by the osmium tetroxide. (E) Calcified calcibacteriocytes, released to the sponge mesohyle; scale bar 1 μm. (F) Sponge larva with dividing calcibacteria, transmitted from a maternal calcibacteriocyte (cbc): larval cilia (c and arrows); larval pseudoepithelial cell; (e) scale bar 2 μm.

**Figure 4:**
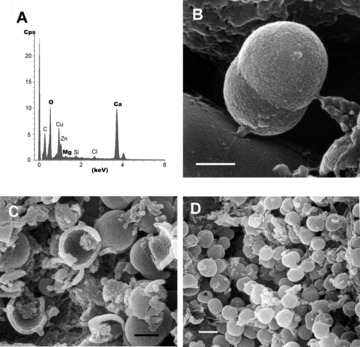
SEM micrographs and EDX of calcibacteria. (A) Energy dispersive X-ray Analysis (EDX) of the calcareous coat. (B) Dividing calcibacteria entrapped within the calcareous coat (SEM); scale bar 600 nm. (C) Broken calcibacteria showing the thin calcareous coat and the inner organic matter (SEM); scale bar 500 nm. (D) High density of calcibacteria released from broken calcibacteriocytes after squeezing the sponge (SEM); scale bar 1μm.

Viewed through electron microscopy, the calcibacteria showed loose inner material and appeared to be devoid of a typical cell wall; they were instead surrounded by a 100-nm-thick calcareous coat. Their organic content was arranged in a peripheral ring bounded by the bacterial membrane and an inner nucleoid (Fig. 3A, B). The electron microscopy observations indicated that recently replicated calcibacteria were not calcified and were able to replicate within the calcibacteriocyte vacuoles (Fig. 3C); subsequently, they secreted a calcareous coat consisting of 20–30 nm nanospherules (Fig. 3D, E). Finally, the cytoplasmic content was progressively reduced, and only the empty spherules remained (Fig. 3E). EDX microanalysis showed that the coat did not contain P, but Mg and Ca in a ratio corresponding to low-Mg calcite crystallization (Fig. 4A). Interestingly, the calcification process can be initiated even before bacterial division is complete (Fig. 4B). Calcibacteriocytes completely filled with calcibacteria were concentrated at the sponge periphery, conferring a whitish tinge to the sponge surface (Fig. 1D), where they lysed and released the calcibacteria to the mesohyl near the sponge surface.

We observed the vertical transmission of calcibacteria during the reproductive cycle of *H. columella*. The maturation of sponge embryos involves the incorporation of maternal cells (nurse cells). Maternal calcibacteriocytes were observed surrounding an embryo (Fig. 1E) and were phagocytized by the growing embryo, as it is reported for typical nurse cells. Once engulfed, the calcibacteriocytes disintegrated and released the calcibacteria into the embryo's mesohyl (Fig. 3F). The noncalcified calcibacteria replicated and were phagocytized by ameboid (archeocyte-like) embryonic cells, after which calcification proceeded. Thus, the sponge larvae contained abundant calcibacteria upon release, and recently settled sponges (rhagons) already exhibited an accumulation of calcibacteriocytes in their surface tissues (Fig. 1F).

## Discussion

To the best of our knowledge, this is the first report of intracellular calcifying bacteria living in symbiosis with marine invertebrates. Vacelet et al. (1987) first reported the presence of irregular calcareous bodies in the sponge *Hemimycale columella* but did not ascertain their nature. Irregular calcareous bodies similar to those reported in *H. columella* have also been described from the tropical Spirophorida *Cinachyrella alloclada* (Rützler and Smith 1992), which suggests the presence of calcibacteria in the later species as well.

The genus *Hemimycale* belongs in the order Poecilosclerida that mainly harbors “low bacterial abundance” sponges (Hentschel et al. 2003). However, our findings show that a sole morphotype of calcibacteria contributed up to 60% of the consortium weight in *Hemimycale* spp., whereas the diverse bacterial assemblages that characterize “high microbial abundance” sponges only accounted for up to 38% of the sponge volume (Vacelet 1975). This result supports the recently manifested need to reevaluate earlier sponge categorizations according to the abundance of their associated bacteria (Thacker and Freeman 2012).

Ankyrins, adhesion-related proteins (ARP) and tetratricopeptide repeat domain-encoding proteins (TPR), which may allow sponge cells to discriminate between food and bacterial symbionts, have been detected in the genome of several sponges harboring bacterial symbionts (Thomas et al. 2010; Liu et al. 2011), which denotes the capacity of sponge cells to identify potential symbionts. Whether these proteins are expressed in all sponge cells or are specific to archeocytes remains unknown. *Hemimycale* calcibacteria were contained in archeocyte-like cells, but they were absent from the more specialized sponge cells: choanocytes (water pumping), collencytes (collagen secretion), sclerocytes (spicule formation), and pinacocytes (pseudoepithelia formation). Archeocytes are pluripotent ameboid cells that move across the sponge mesohyl, phagocytizing cell debris and bacteria (Boury-Esnault and Rützler 1997) and are involved in immunologic processes (Sabella et al. 2007). Due to these abilities, archeocytes may detect “foreign” material in the sponge tissues, including microbes, and are thus particularly suitable cells for developing bacterial symbiosis. Once phagocytized, the microbes are recognized as a food and undergo digestion in archeocyte lysosomes or are identified as “potential partners” and kept alive and allowed to divide within the vacuole.

The phylogenetic identification of the *Hemimycale* calcibacterium is underway. A 16S rRNA gene tag-pyrosequencing of *H. columella* tissue has revealed that the most frequently recovered sequence (65%) was a representative of the Alphaproteobacteria class (authors’ current research), within which mitochondria and many parasitic and symbiotic bacteria of invertebrates fit. These abundant bacteria may correspond to the *Hemimycale* calcibacteria, which is in agreement with the fact that the Bacteroidetes, Betaproteobacteria, and Actinobacteria probes did not gave positive signal in the CARD FISH assays on the sponge tissue. However, most in-depth studies, including in situ hybridization with other specific molecular probes, need to be conducted to confirm their taxonomic identity.

In contrast to other endosymbiotic bacteria previously reported in sponges, the calcibacteria of *Hemimycale* lack bacterial walls, show scant cytoplasmic material, and are contained in cytoplasmic vacuoles, as has been reported for some obligate endosymbionts of other invertebrates (e.g., Baumann et al. 2000; Shigenobu et al. 2000; Pérez-Brocal et al. 2006). These characteristics suggest evolutionary adaptations for living within eukaryotic cells. Overall, the evidence presented in this work suggests a consistent symbiotic sponge–calcibacteria relationship for the following reasons: (i) calcibacteria are present and abundant in all representatives of the host taxon (genus *Hemimycale*) reported from several oceans under distinct ecological conditions, (ii) there is a lack of obvious adverse effects in either symbiont or host, and (iii) calcibacteria are vertically transmitted to sponge progeny. This symbiosis likely has profound implications for the sponge biology, with positive, predictable effects. The improvement of sponge fitness through a series of beneficial functions, such as Ca^2+^ detoxification and supplementary skeletal formation, is the most obvious purported benefits.

Additional advantageous functions derived from calcibacteria can be proposed. *Hemimycale* are not edible by fish, sea urchins, or other benthic predators (Becerro et al. 1997), although the siliceous skeleton, which has been reported to deter fishes from consuming some sponges (Burns and Illan 2003), is poorly developed. *Hemimycale columella* also seems to be weakly defended chemically, as its secondary metabolites only display antimitotic activity (Becerro et al. 1997). The enormous density of calcibacteria at the sponge surface is likely to contribute to the reported deterrence against potential predators. However, the putative antipredatory role of calcibacteria and the environmental conditions preventing or enhancing their proliferation deserve future ecological investigations.

On the other hand, the swift proliferation of the bacteria and their consequent accumulation in the host tissues suggest potential interference with the sponge's biological functions. Curiously, calcibacteria accumulation does not seem to harm the sponge in any appreciable way. In fact, *Hemimycale* sponges grow faster (according to the authors’ current research) than other demosponges in the same habitat (e.g., Turon et al. 1998; Blanquer et al. 2008; De Caralt et al. 2008), perhaps assisted by the supplementary biomass of replicating calcibacteria. Calcification within the calcibacteriocyte vacuoles could be a mechanism that maintains bacterial replication below a threshold that could damage the sponge. Furthermore, calcibacteriocytes accumulate at the sponge periphery and release calcibacteria to the sponge periphery in a similar way to that reported for spherulous or excretory sponge cells (Uriz et al. 1996). Together, these mechanisms may prevent calcibacteria from harming the sponge and, as a by-product, allow for the formation of a rudimentary skeletal cortex on the sponge surface.

The extent of the sponge–calcibacteria association remains unknown, but this symbiosis may be more common than presently thought. The customary use of nitric acid to remove the organic matter for spicule cleaning dissolves calcium carbonate and may have prevented their detection in typical taxonomic studies. Calcibacteria have also recently been found in the poecilosclerid *Crella cyatophora* from the Red Sea (authors’ current research) and, as stated above, may be present in the Spirophorida *Cinachyrella alloclada* (Rützler and Smith 1992). If calcibacteria occur in at least two distant Orders of demosponges (Poecilosclerida and Spirophorida), and might have been overlooked in others, then a phylogenetic study of these bacteria may give insights on the evolutionary origin of this association and possibly trace it back to a common sponge ancestor. This scenario might support the potential role of endosymbiotic bacteria on the origin of calcification processes in basal metazoans, backing implications of previous studies suggesting horizontal transfer of bacterial calcification genes to sclerosponges (Jackson et al. 2011). As in sclerosponges, the siliceous skeleton of *Hemimycale* has been reduced considerably compared with taxonomically close species, perhaps to accommodate the extensive calcareous sclerites. However, we have shown here that unlike in sclerosponges, in which calcification is ultimately produced by the sponge (Jackson et al. 2007, 2011), the calcium carbonate skeleton in *Hemimycale* is a direct result of the associated bacteria.

The elimination of excess cellular Ca^2+^, which is incompatible with life, has been proposed as one of the evolutionary forces triggering a variety of mineral exoskeletons in marine invertebrates (Simkiss 1977; Brennan et al. 2004). Relatively early in animal life, the Ca^2+^ ion became a messenger of basic cell functions, which forced cells to maintain intracellular calcium ions at concentrations several orders of magnitude lower than those in the ambient seawater (William 2000). Thus, once cells were threatened due to calcium overshoot, natural selection would have acted to fix mechanisms for either actively reducing intracellular Ca^2+^ concentrations or tolerating the intracellular precipitation of calcium salts (Lowenstam and Margulis 1980). It has been hypothesized that calcium extrusion first evolved in prokaryotes to protect magnesium enzymes and to prevent phosphates from precipitating (Hoehler et al. 2001). According to the widely accepted “seriated endosymbiosis theory” (Margulis 1981), it is conceivable that bacteria also played a role in calcium regulation in early cell consortia. However, calcifying endosymbiotic bacteria have not been reported in metazoans until now.

The *Hemimycale* species certainly have the potential to serve as exceptional living models for the experimental study of bacteria-mediated calcification in metazoans and the biological interactions between calcibacteria and eukaryotic cells.
